# Seroprevalence of TORCH Viral Agents in Pregnant Women in Turkey: Systematic Review and Meta-Analysis

**DOI:** 10.3390/pathogens14010037

**Published:** 2025-01-06

**Authors:** Elmas Pinar Kahraman Kilbas, Ihsan Hakki Ciftci, Imdat Kilbas, Hande Toptan

**Affiliations:** 1 Department of Medical Laboratory Techniques, Health Services Vocational School, Fenerbahce University, 34758 Istanbul, Turkey; elmspnrkk@gmail.com; 2 Department of Medical Microbiology, Faculty of Medicine, Sakarya University, 54100 Sakarya, Turkey; handetoptan@sakarya.edu.tr; 3 Medical Microbiology Doctorate Program, Institute of Health Sciences, Istanbul University, 34093 Istanbul, Turkey; imdtklbs@gmail.com

**Keywords:** Rubella, Cytomegalovirus, Hepatitis B, Hepatitis C, Herpes Simplex Virus

## Abstract

Rubella Virus, Cytomegalovirus (CMV), Herpes Simplex Virus-2 (HSV-2), Hepatitis B (HBV) and Hepatitis C virus (HCV) can cause serious fetal disease. The seropositivity rates of these agents vary among countries and geographic regions. This study aimed to analyze the prevalence rates and diagnostic methods used in studies investigating the seroprevalence of viral pathogens in the TORCH group among pregnant women in Turkey between 2005 and 2024. A systematic search was conducted using electronic databases between January 2005 and January 2024. A total of 60 studies meeting the inclusion criteria were included. Data quality control was assessed using the Joanna Briggs Institute guideline prevalence studies checklist. Heterogeneity was measured using the I-squared (I^2^) statistic in the Comprehensive Meta Analysis (CMA) program. The average seropositivity rates for Rubella, CMV, HSV-2, HBV and HCV in Turkey were determined as 91.18%, 94.81%, 35.52%, 1.66% and 0.25%, respectively. When the diagnostic methods were examined, it was determined that ELISA and ECLIA methods were used most frequently. The seropositivity of the agents did not show statistically significant differences according to the year periods, geographical regions and age of the patients (*p* > 0.05). The highest prevalence rates of Rubella and HSV-2 in pregnant women were reported in the Mediterranean region, the highest prevalence rates of CMV and HCV in the Southeastern Anatolia region and the highest seroprevalence of Anti HBs in the Marmara region. The results of this study support the necessity of increasing public awareness in the control of fetal infection caused by TORCH viral agents, prenatal screening, vaccination for Rubella and HBV and compliance with hygiene conditions for agents such as CMV, HSV-2 and HCV. The results of this study highlight the need to increase public awareness on prenatal screening for the control of fetal infection caused by all TORCH viral agents, vaccination for Rubella and HBV and compliance with hygiene conditions for agents such as CMV, HSV-2 and HCV.

## 1. Introduction

Viral infections that develop during pregnancy may be asymptomatic or may cause serious infections in the fetus and miscarriage. The TORCH test is an acronym for *Toxoplasma gondii*, “Others (Varicella Zoster (chickenpox) Hepatitis B (HBV), Hepatitis C (HCV), Leptospirosis, Epstein Barr Virus (EBV), Human Immunodeficiency Virus (HIV), Human Parvovirus B19)”, Rubella, Cytomegalovirus (CMV) and Herpes Simplex Virus (HSV) [1]. TORCH agents are a significant concern in pregnant women due to the risks of miscarriage, congenital anomalies in the fetus and high treatment costs. There are many sensitive and specific tests used for serological diagnosis of these agents [2]. Some guidelines for routine serological screening for TORCH agents in pregnancy recommend this, while others have stated that screening is unnecessary. It is recommended that the screening decision be made taking into account the seroprevalence rates in the region and the number of patients susceptible to infections [3].

Rubella is a worldwide pathogen that can cause premature birth, low birth weight, stillbirth, congenital rubella syndrome and miscarriages in the first trimester of pregnancy. Rubella infection is severe during pregnancy, especially in the first trimester [3]. It can cause congenital birth defects and mental retardation in the fetus. The risk of congenital malformations in the newborn is 50%, 25% and 17% in the first, second and third months, respectively [4,5]. The number of rubella infection cases worldwide was reported as 3.3 million in 2015, and it is stated that congenital rubella syndrome developed in more than 100,000 of these cases [6].

CMV can be transmitted congenitally from mother to baby during pregnancy. It affects approximately 60% of women of childbearing age in developed countries and 90% in developing countries [1]. In developed countries, CMV seroprevalence in women of childbearing age ranges from 50% to 85% [7]. Primary maternal infection occurs in 1–4% of susceptible women, and reactivation may occur in approximately 10% of seropositive women. CMV infection in pregnant women is often not recognized. However, it may present as a mild febrile illness with nonspecific symptoms such as fatigue, myalgia, rhinitis, pharyngitis and headache [1].

A mother infected with HSV during pregnancy may pass it congenitally to her baby [8]. The risk of fetal transmission is related to factors such as the type of infection (primary or secondary), the immune status of the mother, the mode of delivery and gestational age. The risk of transmission increases in the third trimester, when primary infection develops before neutralizing antibodies are formed [9]. It has been reported that in Asian countries, the HSV-1 seroprevalence rate varies between 70 and 90% and the HSV-2 seroprevalence rate varies between 5 and 30% in different countries [10].

Hepatitis B virus (HBV) and Hepatitis C virus (HCV) infections are important health problems worldwide. HBV and HCV infection can be transmitted congenitally from an infected mother to the fetus via intrauterine, intrapartum or postnatal routes [11]. Liver diseases that occur during pregnancy can lead to serious and progressive clinical conditions [12]. Factors such as high HCV RNA viral load, HIV coinfection, premature rupture of membranes, amniocentesis, episiotomy and invasive fetal monitoring methods are factors that facilitate HCV infection of the fetus [13]. It has been reported that approximately 8% of pregnant women worldwide have HCV infection [14].

The World Health Organization (WHO) estimates that there are 296 million cases of chronic HBV worldwide, with 1.5 million new infections and 820,000 deaths each year. Babies of HBV carriers develop chronic liver disease at an earlier age [15,16]. The positivity rates of hepatitis B surface antigen (HBsAg), a marker of active HBV infection, in pregnant women vary from region to region. While this rate was reported as 1.1% in a study conducted with asymptomatic pregnant women in Northern India, it was reported as 4.9% in a meta-analysis study conducted with pregnant women in Africa [17,18]. Therefore, HBsAg screening is recommended in the first trimester of pregnancy [19].

Another factor that is of concern in terms of health problems it creates during pregnancy is Zika Virus (ZIKV). This virus has severely affected Brazil and almost all countries in the Americas between 2015 and 2016 [20]. There is a risk of vertical transmission of ZIKV infection during pregnancy, which can lead to congenital infection and babies can be born with Congenital Zika Syndrome (CZS) [21,22]. According to the Centers for Disease Control and Prevention (CDC) report published on 15 May 2024, there is no ZIKV outbreak in any country today. Although it is not a factor of concern in Turkey, it should be kept in mind in the differential diagnosis of viral infections seen in pregnant women [23].

The aim of this study is to compare the prevalence rates reported in studies examining the seroprevalence of TORCH group viral pathogens in pregnant women in Turkey between 2005 and 2024 in terms of different parameters such as geographical region, age and diagnostic methods used. Since these agents during pregnancy carry risks such as congenital infection, abortion and serious health problems in newborns, screening for TORCH group viral pathogens is of great importance for maternal and infant health. The study aims to provide information on which regions and which methods are more effective for early diagnosis and intervention by determining the prevalence rates of these agents.

## 2. Materials and Methods

### 2.1. Protocol

This study was planned based on the Preferred Reporting Items for Systematic Reviews and Meta-Analyses (PRISMA) procedure rules [24]. The PROSPERO registration number of the study is CRD42024573843.

### 2.2. Literature Search

A systematic search was conducted using PubMed, Medline, Embase, Web of Science, EBSCO, Scopus, Turk Medline and Google Scholar electronic databases between January 2005 and January 2024. The search was conducted using words such as “seroprevalence of viral pathogens during pregnancy”, “Rubella prevalence during pregnancy”, “CMV prevalence during pregnancy”, “HBV prevalence during pregnancy”, “HCV prevalence during pregnancy” and “HSV prevalence during pregnancy” in English and Turkish (Appendix A).

The keywords used in the study were scanned by three independent authors, their titles and abstracts were scanned in terms of relevance and, after a detailed evaluation, original articles that met the inclusion criteria were included in the study. The total number of studies found according to the keywords determined in the databases was 6332, and the full texts of 1914 of them were accessed. After elimination according to the exclusion criteria, a total of 123 research articles were included in the study.

### 2.3. Inclusion and Exclusion Criteria

Research articles published in national and international peer-reviewed journals, which full texts could be accessed and were defined at the species level and made the distinction between IgM and IgG, were included in this meta-analysis. In addition, studies reporting antibody test results of at least 100 pregnant women, publications published between 2005 and 2024, which publication language was Turkish or English and which quality assessment score was above 7 points, were deemed appropriate to be included.

Publications that could not be accessed in full text, that did not have a species-level definition, that had inconsistencies and uncertainties in their data, that were published in languages other than English or Turkish, that were published before 2005, that could not be accessed in full text, that were not conducted with patient data in Turkey, that did not make an IgM/IgG distinction, that were not research articles and that had a quality assessment score below 7 points were eliminated.

### 2.4. Quality Control of Data

The quality of the studies was assessed by the authors using the Joanna Briggs Institute guideline prevalence studies checklist. The checklist consists of nine questions that the reviewers answered for each study. A “Yes” answer to the questions was evaluated as 1 point. Thus, the total score of each study ranged from 0 to 9. Studies with a score of 4 to 6 were considered medium quality, and those with a score of 7 to 9 were considered high quality [25]. Publications with a total score below 7 were eliminated, and all studies with a score between 7 and 9 were evaluated within the scope of the meta-analysis.

### 2.5. Data Analysis

During the literature review process, titles and abstracts were examined and full texts of the studies were accessed. Microsoft Excel spreadsheets were prepared for data collection, and these tables included the first author’s surname, publication year, location of the study, sample size and identification methods used in the studies. Data analysis was performed using SPSS software (IBM SPSS Statistics, Version 25.0; IBM Corp., Armonk, NY, USA).

A random effects model was used for the meta-analysis. Statistical significance was determined using *p* < 0.05 and a 95% confidence interval. Heterogeneity was measured using the I-squared (I^2^) statistic. To assess publication bias, a funnel plot was created with Comprehensive Meta-Analysis (CMA) ver. 3.3 software (Biostat, Englewood, CO, USA), and the Egger test was applied. A sensitivity analysis was performed to assess the impact of a single study on the overall estimate.

In order to determine the potential causes of heterogeneity, meta regression analysis was performed. In this analysis, the relationship between independent variables (publication quality score, sample size, region, method and year) and a dependent variable (effect size) was evaluated. The regression model was established to determine how the variation in effect size is related to one or more potential moderators. The extent to which the factors explaining heterogeneity explain the variability in effect size is reported with the R-squared (R^2^) value. In the analyses, the effect of each subgroup was tested statistically, and the significance level was determined as *p* < 0.05.

## 3. Results

### 3.1. Characteristics of the Studies

**Figure 1:**
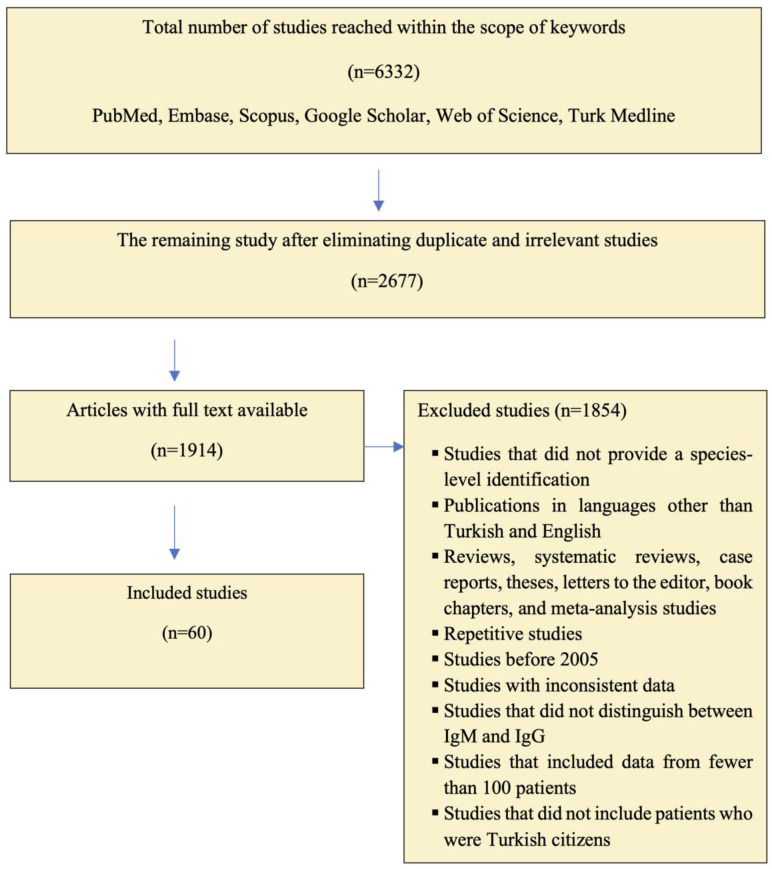
Studies excluded and included in the study based on the search criteria (PRISMA flow chart).

As a result of the database search, a total of 6332 studies published between 2005 and 2024 were identified. As a result of the evaluation of the studies reporting seroprevalence according to the inclusion and exclusion criteria, a total of 60 studies were determined to meet the inclusion criteria: 34 for Rubella, 32 for CMV, 6 for HSV-2, 25 for HBV and 17 for HCV (Figure 1).

Since sufficient data were not found for HSV-1, only articles reporting HSV-2 were evaluated. The included publications were analyzed by dividing them into two periods as 2004–2015 and 2016–2024. The same publications were divided into seven groups according to the geographical regions of Turkey: Eastern Anatolia, Black Sea, Southeastern Anatolia, Central Anatolia, Marmara, Mediterranean and Aegean Regions. The characteristic features of all included publications are shown in Table 1a–e.

It was observed that the seroprevalence of Rubella, CMV and HCV was lower in the period between 2016 and 2024, but this difference was not statistically significant (*p* = 0.41, *p* = 0.47 and *p* = 0.06; *p* > 0.05). It was determined that the prevalence of HSV-2 and HBV increased over the years, but this difference was not statistically significant (*p* = 0.82, 0.43; *p* > 0.05).

A total of 142,438 patients’ data were examined for IgG from the studies reporting the prevalence of Rubella included in the meta-analysis, and IgG was positive in 129,877 (91.18%) patients, and a gray zone (intermediate value) was detected in 615 (2.08%) patients. For IgM, 153,591 patients were included. IgM was positive in 1380 (0.89%) patients, and a gray zone was in 271 (0.17%) patients. It was observed that the regions reporting the most data for Rubella were the Aegean and Marmara regions. In addition, no statistically significant difference was found between Rubella seropositivity and age (*p* = 0.532; *p* > 0.05).

In the studies included in the meta-analysis, data from a total of 87,743 patients were included for CMV IgG, and it was found that 83,196 (94.81%) of the patients were IgG-positive and 14 (0.01%) were in the gray zone. For IgM, 110,003 patients were included, and it was found that 1853 (1.68%) were IgM-positive and 218 (0.19%) were in the gray zone. It was determined that the regions reporting the most data for CMV were the Central Anatolia and the Aegean region. In addition, no statistically significant difference was found between CMV seropositivity and age (*p* = 0.604; *p* > 0.05).

A total of 4465 patient data were examined for HSV-2 IgG in the included studies, and 1586 (35.52%) of the patients were found to be positive, and 4328 patient data were examined for IgM, and 132 (3.04%) were found to be positive. It was seen that the most data reporting for HSV-2 was made from the Central Anatolia region.

When the HBV studies included in the meta-analysis were examined, 10,002 (1.66%) out of 602,198 patients were found to be positive for HbsAg, and 48,601 (27.46%) out of 166,014 patients were found to be positive for Anti-HBs. It was found that Anti-HBs seroprevalence did not show any statistically significant difference according to the years (*p* = 0.306; *p* > 0.05). It was determined that the regions reporting the most data for HBV were the Marmara and Black Sea regions. In addition, no statistically significant difference was found between HbsAg seropositivity and age (*p* = 0.49; *p* > 0.05).

For Anti-HCV, 304,342 patient data were examined, and positivity was reported in 786 (0.25%) of the patients. It was determined that the region reporting the most data for HCV was the Marmara region. No statistically significant difference was found between HCV seropositivity and age (*p* = 0.107; *p* > 0.05).

When the diagnostic methods were examined, it was found that the ELISA method was used most frequently for the diagnosis of Rubella, CMV, HSV-2 and HCV at rates of 53.13%, 38.24%, 83.3% and 58.82%, respectively, and the ECLIA method was used most frequently for the diagnosis of HBV (48%) (Table 1a–e).

### 3.2. Subgroup Analyses

**Figure 2:**
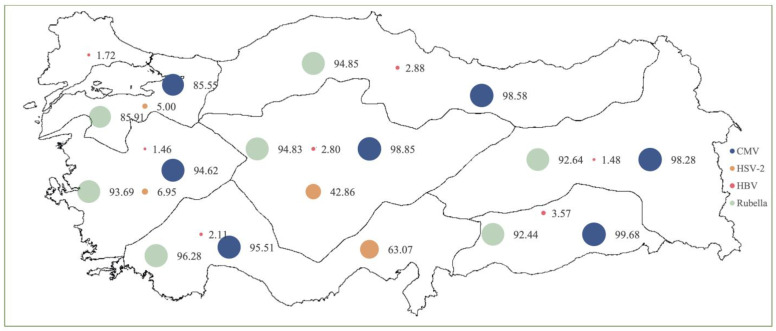
Seroprevalence rates of viral agents according to geographical regions (%).

The highest prevalence rates of Rubella in pregnant women were in the Mediterranean (96.28%), Central Anatolia (94.83%) and Black Sea (94.85%) regions, respectively (*p* > 0.05). The highest prevalence rates of CMV were found in the Southeastern Anatolia (99.68%), Central Anatolia (98.85%) and Black Sea (98.59%) regions, respectively (*p* > 0.05). The highest prevalence of HSV-2 was found in the Mediterranean (63.08%) and Central Anatolia (42.86%) regions (*p* > 0.05). The highest seroprevalence of HbsAg was reported in the Southeastern Anatolia region (3.57%), while the highest prevalence rates of HCV were reported in the Southeastern Anatolia (0.73%) and Central Anatolia (0.44%) regions (*p* > 0.05) (Table 2 and Figure 2).

### 3.3. Heterogeneity

The data for each outcome are summarized in funnel plot graphs. We found that 32 studies on CMV had high heterogeneity, and the random effect size was calculated as 0.98 (I^2^ = 99.71%). Additionally, 34 studies on Rubella had high heterogeneity, and the random effect size was calculated as 0.94 (I^2^ = 99.83%), while 5 studies on HSV-2 had high heterogeneity, and the random effect size was calculated as 0.09 (I^2^ = 98.65%), 25 studies on HBV had high heterogeneity, and the random effect size was calculated as 0.02 (I^2^ = 99.19%), and 17 studies on HCV had high heterogeneity, and the random effect size was calculated as 0.00 (I^2^ = 92.88%) (Appendix B).

As a result of the meta regression analysis performed to explain the heterogeneity, the R^2^ values calculated for the CMV, HBV and HCV seroprevalence studies were found to be 0.00 (*p* > 0.05). It was observed that the independent variables in the subgroups were insufficient to explain the variation in the effect size of the studies and that different factors may be effective on heterogeneity. However, the R^2^ values calculated for the Rubella seroprevalence studies were found to be 0.03. This model explains 3% of the heterogeneity in effect size. It was determined that sample size was a factor affecting heterogeneity in Rubella studies (*p* = 0.02; *p* < 0.05) (Appendix C). Heterogeneity does not only depend on sample size, but this relationship plays a role in heterogeneity. In this context, it can be said that subgroups such as sample size should be taken into consideration in order to better understand heterogeneity in meta-analyses.

## 4. Discussion

Rubella, CMV, HSV, HBV and HCV are important public health problems that can be seen in every age group in the world and in our country and can cause prenatal and perinatal infections in pregnant women [32]. These agents can cause miscarriage, premature birth and congenital malformations in pregnant women. Determining the prevalence of these viral infectious agents in the TORCH group plays an important role in taking the necessary precautions to reduce the effects of infections in pregnant women [1].

Rubella is one of the most common viral infection agents that cause fetal infection. It can lead to congenital rubella syndrome, which occurs with vision and hearing problems, mental retardation, microcephaly and cardiac diseases in the fetus [85]. In our study, the average Rubella IgG positivity in pregnant women was determined as 91.18%. In a meta-analysis study that included studies conducted on pregnant women in Sub-Saharan Africa, IgG positivity was reported as 89% [5]. In another meta-analysis study examining Rubella seroprevalence in pregnant women, the Rubella IgG seroprevalence rates in pregnant women were reported as 93.47% [86]. As a result of a meta-analysis study conducted in 2017, Rubella seropositivity was reported as 86.6–98.6% in the World Health Organization European Region [87]. The data of our study are similar to the literature. Since the seroprevalence rates are above 90%, all seronegative women should be vaccinated before pregnancy planning in order to prevent fetal anomalies [32].

The overall prevalence rate of congenital infection caused by CMV worldwide is approximately 0.5–2%, and it is the most common congenital viral infection [88]. In the studies included in this meta-analysis, the average CMV IgG seropositivity rates were found to be 94.81%. In a meta-analysis study conducted on pregnant women in Turkey, the CMV IgG seropositivity seroprevalence rates were reported as 97.98%. Ozdemir et al. (2016) reported CMV IgG seropositivity rates as 87.8–100% in a multicenter study [42]. Wang et al. (2023) reported that the seropositivity rates for CMV-IgG were 73.8% in their meta-analysis on TORCH seroprevalence in women of childbearing age [89]. Since the CMV vaccine is not yet available, protective measures such as hand washing and avoiding contact with body fluids such as urine and saliva of young children are important in pregnant women.

HSV can cause serious infection conditions with neurological involvement in newborns born to mothers with primary infection. In the studies included in this meta-analysis, the average seroprevalence rates were found to be 35.52% for HSV-2 IgG. It was observed that the most data reporting for HSV-2 seroprevalence in pregnant women were from the Central Anatolia region. Radoi et al. (2024) reported the HSV-1 seroprevalence rate as 84.96% and HSV-2 seroprevalence as 12.43% in pregnant women in Romania between 2019 and 2022 [90]. In the studies included in the meta-analysis, it was seen that the highest seroprevalence rate was reported in Ankara at 81.31%, and the lowest rate was reported in Konya at 4.2%. Since it is known that the prevalence of HSV is higher in developed regions, these rates are parallel to the literature. Placental transmission of HSV from mother to fetus is quite rare. Since transmission is usually transmitted during vaginal delivery, cesarean section is recommended to prevent neonatal infections in pregnant women with confirmed HSV. Therefore, the importance of HSV seroprevalence in pregnant women is uncertain, and many countries do not recommend routine screening for pregnant women [91].

HBV seroprevalence is high in low- and middle-income countries. In order to prevent perinatal transmission, prenatal screening should be performed in all pregnant women. When the studies on HBV included in our study were examined, the average HbsAg positivity was found to be 1.66% and Anti-HBs positivity was 27.46%. It was determined that the regions reporting the most data for HBV were the Marmara and Black Sea regions. In meta-analysis studies conducted to investigate the frequency of HBV infection in pregnant women in India and Africa, the HBsAg positivity rate was reported as 1.6% and 6.77%, respectively [92,93]. Oner et al. (2021) reported HBsAg positivity in 1.5% of 1361 pregnant women [80]. Furuncuoglu et al. (2016) reported the HBsAg seropositivity as 4.24% in their study conducted between 1995 and 2015 and found that this rate decreased over the years [94]. In our study, it was found that HBsAg seroprevalence did not show any statistically significant difference according to years and geographical regions. However, the highest HBsAg rates were found in the Southeastern Anatolia (3.57%) and Black Sea (2.88%) regions. The differences in distribution between regions may be due to multiple factors, such as piercing and tattooing habits, vaccination, pre-pregnancy blood collection status and the prevalence of immigrants. While the overall rate of active HBV infection in Turkey is similar to some studies in the literature, it was found to be lower compared to the rates reported in underdeveloped regions such as the African continent or the Southeastern Anatolia region of our country. In addition, no statistically significant difference was found between HBsAg seropositivity and age. The reason for this was that the mean ages reported in all the articles were close to each other. In most of the included studies, there was no data on the vaccination status of pregnant women, HBeAg and HBV viral load that should be done to start antiviral treatment in pregnant women with active HBV infection.

In the studies included in this meta-analysis, Anti-HCV positivity was detected in an average of 0.25% of the patients. Although HCV seroprevalence rates did not show a statistically significant difference over the years, it was observed that the prevalence rate decreased over the years [70]. In a study they conducted in Bolu, they reported Anti-HCV positivity in 0.5% of pregnant women. Oner et al. (2021) determined the HCV seroprevalence as 0.15% in 1361 pregnant women [80]. In addition, our country is in the low endemicity group in terms of HCV. Therefore, the necessity of routine screening of pregnant women for Anti-HCV to prevent vertical transmission of HCV is controversial and is not considered cost-effective [95]. Instead, it is recommended at level 1B of evidence that women of childbearing age who are diagnosed with HCV infection should be treated with direct-acting antivirals before pregnancy by performing Anti-HCV screening during pregnancy planning [96]. It is reported in the literature that factors such as dental treatment, medical abortion, delivery and hospitalization are important risk factors [97]. Therefore, effective sterilization measures should be taken, especially during the application of invasive procedures in pregnant women.

This study has some limitations. The first of these is that studies reporting HBV and HCV in pregnant women could not be evaluated due to insufficient data on invasive procedure history such as tattoo presence, piercing use, etc. In addition, the immunization information of patients could not be obtained in most studies providing HBV data. Data on other viruses that can cause serious infections in pregnant women (Parvovirus B19, HSV-1, Epstein-Barr virus, etc.) could not be included in the meta-analysis due to the small number of studies.

## 5. Conclusions

It was found that the seroprevalence of viral agents examined within the scope of the TORCH panel in pregnant women did not show a significant change over the years. As a result of this study, it was seen that the prevalence rates determined were similar to the results of other meta-analyses in the literature. Although there was no statistically significant difference between the regions, the highest prevalence rates of Rubella and HSV-2 in pregnant women were reported in the Mediterranean region, the highest prevalence rates of CMV and HCV in Southeastern Anatolia, and the highest seroprevalence of HBsAg in the Southeastern Anatolia region. The results of this study revealed the necessity of increasing public awareness on prenatal screening; vaccination for Rubella and HBV and compliance with hygiene conditions for agents such as CMV, HSV and HCV in order to control fetal infection caused by TORCH viral agents. The lack of a guideline for routine screening of these microorganisms before and during pregnancy in Turkey was determined as a deficiency. Since it is known that seroprevalence rates vary according to countries and geographical regions, reporting these rates is important in terms of maturing screening schemes and raising awareness of women about ways to protect themselves from these infections.

## Figures and Tables

**Table 1:** (**a**–**e**): Characteristics of studies included in the meta-analysis [26,27,28,29,30,31,32,33,34,35,36,37,38,39,40,41,42,43,44,45,46,47,48,49,50,51,52,53,54,55,56,57,58,59,60,61,62,63,64,65,66,67,68,69,70,71,72,73,74,75,76,77,78,79,80,81,82,83,84].

a. Rubella										
Study Name	Study Design		Region	Age Range (Average)	Sample Size	IgG Positivity (n)			Prevalence (%)	Diagnostic Method
Ocak et al., 2007	R		Mediterranean	16–42 (27)	1652	1570			95.04	ELISA
Uyar et al., 2008	R		Black Sea	17–40 (29)	600	566			94.33	ECLIA
Dundar et al., 2009	R		Eastern Anatolia	16–44 (27)	3416	3151			92.24	ELISA
Efe et al., 2009	R		Eastern Anatolia	15–45 (27)	613	610			99.51	ELISA
Karabulut et al., 2011	-		Aegean	18–40 (29)	1268	1206			95.11	ECLIA
Ozdemir et al., 2011	-		Central Anatolia	18–44 (28)	249	239			95.98	ELFA
Asik et al., 2013	R		Aegean	15–45 (27)	505	465			92.08	ELISA
Keskin and Keskin, 2013	-		Marmara	16–45 (27)	1926	1844			95.74	ELISA
Bakacak et al., 2014	R		Southeast	16–49 (-)	7733	7212			93.26	ELISA
Inci et al., 2014	R		Black Sea	15–45 (27)	1292	1230			95.2	ELISA
Dogan et al., 2014	R		Marmara	16–48 (28)	1641	1571			95.73	ELISA
Karacan et al., 2014	-		Marmara	18–44 (30)	1258	1196			95.07	ELFA
Kiris Satilmis et al., 2014	R		Marmara	15–50 (-)	804	756			94.03	ELFA
Parlak et al., 2015	R		Eastern Anatolia	-	416	360			86.54	ELISA
Aynioglu et al., 2015	R		Black Sea	17–46 (29)	868	854			98.39	ECLIA
Numan et al., 2015	R		Marmara	17–47 (30)	1101	1037			94.19	ELISA
Ozdemir et al., 2016	R		Multicenter	-	3231	2976			92.11	ELISA
Simsek et al., 2016	R		Aegean	16–45 (29)	1076	1017			94.52	ELISA
Senturk et al., 2016	R		Black Sea	18–48 (31)	424	398			93.87	ELISA
Akpinar and Akpinar, 2017	R		Mediterranean	-	805	785			97.52	ELISA
Sirin et al., 2017	R, CS		Aegean	18–45 (31)	7189	6721			93.49	ECLIA
Kasap et al., 2017	R		Aegean	18–44 (29)	189	170			89.95	-
Madendag et al., 2018	R		Central Anatolia	18–45 (25)	10,200	9924			97.29	ELISA
Gurlek et al., 2019	R		Black Sea	-	3459	3410			98.58	ECLIA
Cubuk et al., 2020	R		Central Anatolia	-	1135	998			87.93	ELISA
Alacam et al., 2020	R, CS		Marmara	18–48 (28)	8378	2715			32.41	ELISA
Guzel et al., 2020	R		Marmara	17–47 (30)	853	804			94.26	CMIA
Ozel et al., 2021	R, CS		Aegean	- (28)	1172	1147			97.87	ELISA
Dogus Uzun et al., 2022	R		Southeast	- (27)	5370	4920			91.62	ECLIA
Kahraman et al., 2022	R		Black Sea	-	9210	8176			88.77	ECLIA
Kul et al., 2023	R, D, CS		Multicenter	18–54 (31)	56,392	54,368			96.41	-
Ozgur et al., 2023	R		Eastern Anatolia	15–49 (30)	2017	1861			92.27	ELFA
Kinci et al., 2023	R, D, CS		Aegean	16–45 (-)	4978	4621			92.83	ECLIA
Ezer et al., 2023	R		Central Anatolia	16–49 (-)	1018	999			98.13	ELFA
**b. CMV**										
**Study Name**	**Study Design**		**Region**	**Age Range (Average)**	**Sample Size**	**IgM Positivity (n)**		**IgG Positivity (n)**	**Prevalence (%)**	**Diagnostic Method**
Ocak et al., 2007	R		Eastern Anatolia	16–42 (27)	1652	7		1568	94.92	ELISA
Uyar et al., 2008	R		Black Sea	17–40 (29)	600	6		584	97.33	ECLIA
Efe et al., 2009	R		Central Anatolia	15–45 (27)	600	10		597	99.5	ELISA
Oruc et al., 2011	R		Central Anatolia	- (28)	11,360	35		11,189	98.49	ECLIA
Karabulut et al., 2011	-		Black Sea	18–40 (30)	1000	12		987	98.7	ELISA
Ozdemir et al., 2011	-		Marmara	18–44 (28)	249	0		246	98.8	ELFA
Keskin and Keskin, 2013	-		Mediterranean	16–45 (28)	1926	13		1911	99.22	ELISA
Inci et al., 2014	R		Aegean	15–45 (27)	1043	17		1028	98.56	ELISA
Bakacak et al., 2014	R		Marmara	16–49 (-)	5467	175		5432	99.36	ELISA
Dogan et al., 2014	R		Marmara	16–48 (28)	1769	14		1756	99.27	ELISA
Karacan et al., 2014	-		Eastern Anatolia	18–44 (30)	1258	5		1057	84.02	ELFA
Kiris Satilmis et al., 2014	R		Black Sea	15–50 (-)	804	3		803	99.88	ELFA
Parlak et al., 2015	R		Marmara	-	527	245		527	100	ELISA
Aynioglu et al., 2015	R		Multicenter	17–46 (29)	852	19		833	97.77	ECLIA
Numan et al., 2015	R		Aegean	17–47 (30)	904	4		899	99.45	ELISA
Ozdemir et al., 2016	R		Mediterranean	-	4097	206		3965	96.78	ELISA
Simsek et al., 2016	R		Aegean	16–45 (30)	1048	30		1006	95.99	ELISA
Akpinar and Akpinar, 2017	R		Aegean	-	532	10		497	93.42	ELISA
Sirin et al., 2017	R, CS		Central Anatolia	18–45 (31)	998	14		908	90.98	ECLIA
Kasap et al., 2017	R		Southeast	18–44 (29)	136	1		123	90.44	-
Madendag et al., 2018	R		Black Sea	18–45 (25)	10,200	20		10,017	98.21	ELISA
Gurlek et al., 2019	R		Marmara	-	3404	65		3376	99.18	ECLIA
Cubuk et al., 2020	R		Marmara	-	1123	8		1111	98.93	ELISA
Alacam et al., 2020	R, CS		Aegean	18–48 (28)	3437	72		787	22.9	ELISA
Guzel et al., 2020	R		Southeast	17–47 (30)	309	4		291	94.17	ECLIA
Ozel et al., 2021	R, CS		Black Sea	- (28)	1199	17		1163	97	ELISA
Dogus Uzun et al., 2022	R		Multicenter	- (28)	5370	37		5363	99.87	ECLIA
Kahraman et al., 2022	R		Eastern Anatolia	-	411	89		410	99.76	ECLIA
Kul et al., 2023	R, D, CS		Aegean	18–54 (31)	18,495	646		17,849	96.51	-
Ozgur et al., 2023	R		Central Anatolia	15–49 (30)	1988	9		1987	99.95	ELFA
Kinci et al., 2023	R, D, CS		Mediterranean	16–45 (-)	4539	53		4471	98.5	ECLIA
Ezer et al., 2023	R		Marmara	16–49 (-)	456	7		455	99.78	ELFA
**c. HSV-2**										
**Study Name**	**Study Design**		**Region**	**Age Range (Average)**	**Sample Size**	**IgM Positivity (n)**		**IgG Positivity (n)**	**Prevalence (%)**	**Diagnostic Method**
Duran et al., 2004	-		Multicenter	-	130	18		82	63.08	ELISA
Dolar et al., 2006	CS		Aegean	19–43 (28.1)	300	-		15	5	ELISA
Ozdemir et al., 2011	-		Central Anatolia	18–44 (28.4)	249	0		11	4.42	ELISA
Ozdemir et al., 2016	R		Aegean	-	1382	19		58	4.2	ELISA
Simsek et al., 2016	R		Marmara	16–45 (29.6)	719	14		50	6.95	ELISA
Sert et al., 2019	RCS		Central Anatolia	16–42 (-)	1685	81		1370	81.31	CLIA
**d. HBV**										
**Study Name**	**Study Design**	**Region**	**Age Range (Average)**	**Sample size**	**Positivity (n)**	**Prevalence (%)**	**Sample size**	**Positivity (n)**	**Prevalence (%)**	**Diagnostic Method**
				**HBsAg**			**Anti HBs**			
Tekay and Ozbek, 2006	R	Southeast	-	2335	119	5.1	-	-	-	ECLIA
Dundar et al., 2009	R	Eastern Anatolia	16–44 (27)	3503	78	2.23	3503	568	16.21	ELISA
Kölgelier et al., 2009	R	Southeast	16–49 (29)	677	31	4.58	641	210	32.76	ELISA
Uyar et al., 2009	-	Black Sea	17–45 (28)	2654	56	2.11	40	0	0	ELISA
Guckan et al., 2010	R	Black Sea	16–48 (27)	5540	50	0.9	4752	1410	29.67	ELISA
Yavuzcan et al., 2011	R	Eastern Anatolia	14–53 (28)	473	7	1.48	-	-	-	-
Cicek et al., 2012	R	Southeast	15–49 (28)	56,275	1968	3.5	17,351	3436	19.8	ECLIA
Ozlu et al., 2013	R	Black Sea	- (28)	653	12	1.84	-	-		ELISA
Balik et al., 2013	R	Black Sea	15–47 (28)	5894	338	5.73	4967	1592	32.05	ELISA
Keskin and Keskin, 2013	-	Marmara	16–45 (28)	2900	70	2.41	2900	640	22.07	ELISA
Dogan et al., 2014	R	Marmara	16–48 (28)	1450	18	1.24	1392	366	26.29	ELISA
Dag et al., 2015	R, CS	Central Anatolia	-	8442	293	3.47	3094	1417	45.8	ELISA
Furuncuoglu et al., 2016	R	Marmara	- (23)	7065	114	1.61	7065	877	12.41	ELISA
Kasap et al., 2017	R	Aegean	18–44 (29)	333	5	1.5	333	78	23.42	-
Altuglu et al., 2017	R	Aegean	16–49 (30)	8967	127	1.42	-	-	-	ELISA
Cetin et al., 2018	D, CS	Mediterranean	16–45 (27)	475	10	2.11	-	-	-	CLIA
Sahin et al., 2018	R	Eastern Anatolia	16–46 (28)	2214	22	0.99	2214	381	17.21	ELISA
Cinar Tanriverdi et al., 2019	R	Eastern Anatolia	18–45 (25)	35,295	425	1.2	34,489	9854	28.57	ECLIA
Yalcin Bahat et al., 2019	R	Marmara	- (28)	68,169	1257	1.84	7130	1487	20.86	ELISA
Kale et al., 2020	R	Marmara	-	55,639	822	1.48	11,263	3898	34.61	CLIA
Oner et al., 2021	D	Multicenter	17–49 (28)	1361	21	1.54	1361	708	52.02	ECLIA
Bilman et al., 2021	R	Multicenter	-	204,865	2343	1.14	-	-	-	-
Erin et al., 2021	R	Black Sea	- (27)	10,449	400	3.83	10,449	5177	49.55	ELISA
Gulseren et al., 2022	R	Central Anatolia	-	11,941	255	2.14	-	-	-	CLIA
Hansu et al., 2023	R	Southeast	15–45 (-)	104,629	1161	1.11	53,070	16,502	31.09	-
**e. HCV**										
**Study Name**	**Study Design**	**Region**	**Age Range (Average)**		**Sample size**		**IgG Positivity (n)**		**Prevalence (%)**	**Diagnostic Method**
Tekay and Ozbek, 2006	R	Southeast	-		2066		18		0.87	ECLIA
Dundar et al., 2009	R	Eastern Anatolia	16–44 (27)		3496		2		0.06	ELISA
Kolgelier et al., 2009	R	Southeast	16–49 (29)		183		2		1.09	ELISA
Cicek et al., 2012	R	Southeast	15–49 (28)		13,719		106		0.77	ECLIA
Ozlu et al., 2013	R	Black Sea	- (28)		612		3		0.49	ELISA
Balik et al., 2013	R	Black Sea	15–47 (28)		5681		25		0.44	ELISA
Keskin and Keskin, 2013	-	Marmara	16–45 (28)		2900		4		0.14	ELISA
Dag et al., 2015	R, CS	Central Anatolia	-		8120		36		0.44	ELISA
Kasap et al., 2017	R	Aegean	18–44 (29)		333		1		0.3	-
Altuglu et al., 2017	R	Aegean	16–49 (30)		8865		34		0.38	ELISA
Sahin et al., 2018	R	Eastern Anatolia	16–46 (28)		2214		3		0.14	ELISA
Cinar Tanriverdi et al., 2019	R	Eastern Anatolia	18–45 (25)		9079		6		0.07	ECLIA
Yalcin Bahat et al., 2019	R	Marmara	- (28)		67,760		200		0.3	ELISA
Kale et al., 2020	R	Marmara	-		74,990		159		0.21	CL ELISA
Oner et al., 2021	R	Multicenter	17–49 (28)		1361		2		0.15	ECLIA
Erin et al., 2021	R	Black Sea	- (28)		10,449		10		0.1	ELISA
Hansu et al., 2023	R	Southeast	15–45 (-)		92,514		175		0.19	-

CL: chemiluminescent, CLIA: chemiluminescence immunoassay, CMIA: chemiluminescent microparticle immunoassay, CS: cross-sectional, D: descriptive, ECLIA: electrochemiluminescence immunoassay, ELFA: enzyme-linked fluorescent assay, ELISA: enzyme-linked immunosorbent assay, R: retrospective and RCS: retrospective cohort study.

**Table 2:** Subgroup analyses of the pooled prevalence values of viral agents in pregnant women.

	Region	Number of Studies	95% CI Prevalence	I^2^	*p*
**Rubella**	Mediterranean	2	96.28 [95.04–97.52]	99.83	0.88
	Eastern Anatolia	4	92.64 [86.54–99.51]		
	Aegean	7	93.69 [89.95–97.87]		
	Southeast Anatolia	2	92.44 [91.62–93.26]		
	Central Anatolia	4	94.83 [87.93–98.13]		
	Black Sea	6	94.85 [88.77–98.58]		
	Marmara	7	85.9186 [32.41–95.74]		
	Multicenter	2	94.26 [92.11–96.41]		
**CMV**	Mediterranean	2	95.99 [93.42–98.56]	99.71	0.73
	Eastern Anatolia	3	98.29 [94.92–100]		
	Aegean	6	98.53 [90.44–109.91]		
	Southeast Anatolia	2	99.68 [99.5–99.87]		
	Central Anatolia	4	98.85 [98.21–99.78]		
	Black Sea	6	98.59 [97.33–99.76]		
	Marmara	7	85.55 [22.9–99.88]		
	Multicenter	2	96.64 [96.51–96.78]		
**HSV-2**	Mediterranean	1	63.08 [63.08–63.08]	98.65	0.86
	Aegean	1	6.95 [6.95–6.95]		
	Central Anatolia	2	42.86 [4.42–81.31]		
	Marmara	1	1.67 [1.67–1.67]		
	Multicenter	1	4.2 [4.2–4.2]		
**HBV**	Mediterranean	1	2.11 [2.11–2.11]	99.19	0.00 *
	Eastern Anatolia	4	1.47 [0.99–2.23]		
	Aegean	2	1.46 [1.42–1.5]		
	Southeast Anatolia	4	3.57 [1.11–5.1]		
	Central Anatolia	2	2.80 [2.14–3.47]		
	Black Sea	5	2.88 [0.9–5.73]		
	Marmara	5	1.71 [1.24–2.41]		
	Multicenter	2	1.34 [1.14–1.54]		
**HCV**	Eastern Anatolia	3	0.09 [0.06–0.14]	92.88	0.08
	Aegean	2	0.34 [0.3–0.38]		
	Southeast Anatolia	4	0.73 [0.19–1.09]		
	Central Anatolia	1	0.44 [0.44–0.44]		
	Black Sea	3	0.34 [0.1–0.49]		
	Marmara	3	0.21 [0.14–0.3]		
	Multicenter	1	0.15 [0.15–0.15]		

CI: confidence interval. * *p* < 0.05.

**Figure A1:**
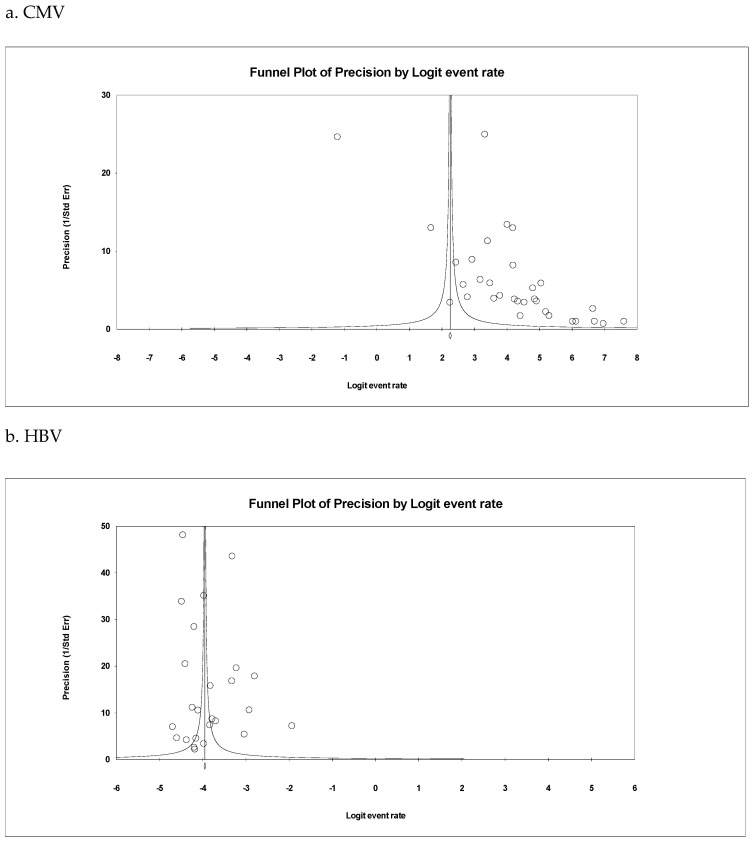

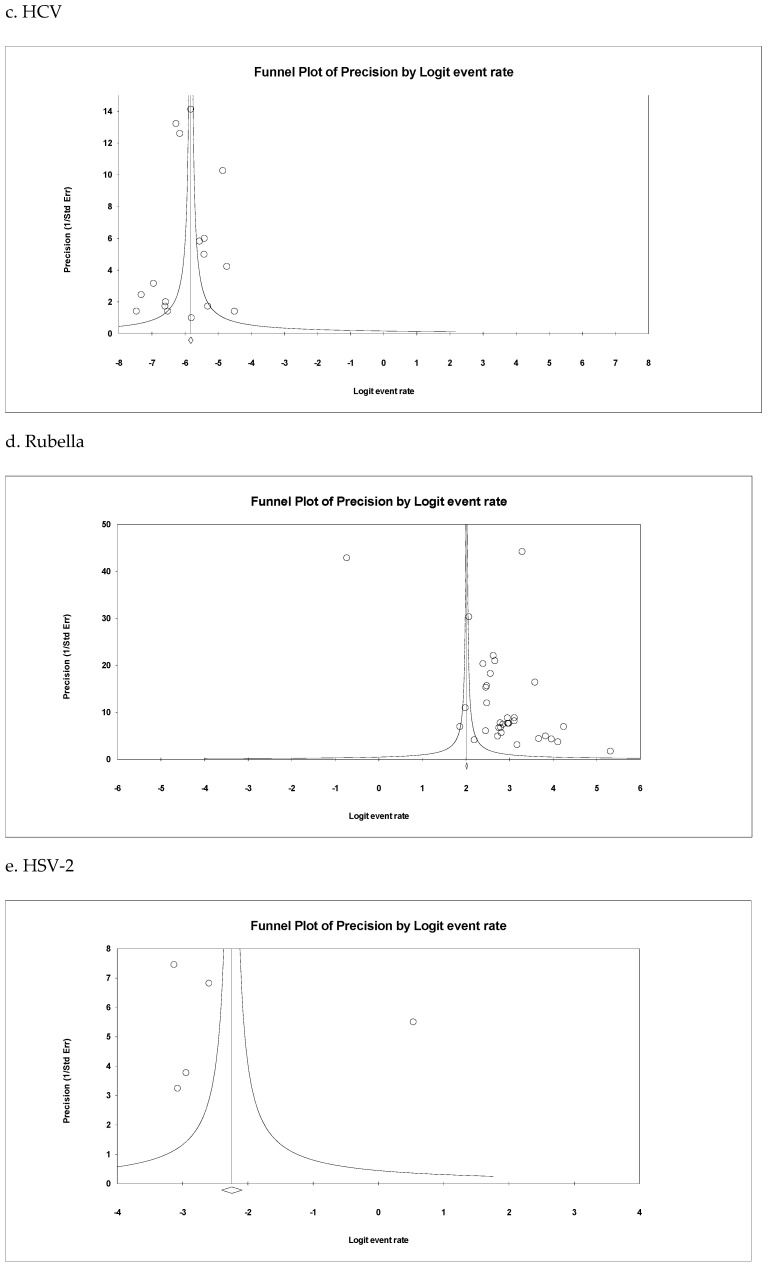
Funnel plot analysis to detect publication bias for CMV, HBV, HCV, Rubella and HSV-2 studies.

## Data Availability

The authors declare that all related data are available from the corresponding author upon reasonable request.

## Appendix A

**Table A1:** Search terms.

Database	Search Terms
PubMed	(“Prevalence” [Mesh] OR “Epidemiology” [Mesh]) AND (“Hepatitis B” [Mesh]) AND (“Hepatitis C” [Mesh]) AND “Cytomegalovirus Infections” [Mesh]) AND “Rubella” [Mesh]) AND “Herpes Simplex Virus” [Mesh]) AND (“Pregnant Women” [Mesh]) AND (“Turkey” [Mesh]).
Google Scholar	“Gebelikte Viral Patojenlerin Seroprevalansı”, “Seroprevalence of Viral Pathogens During Pregnancy”, “Gebelikte Viral Patojenler”, “Viral Pathogens During Pregnancy”, “Prevalence of Rubella During Pregnancy”, “Gebelikte Rubella Prevalansı”, “Gebelikte CMV Prevalansı”, “CMV Prevalence During Pregnancy”, “Sitomegalovirüs”, “Gebelikte HBV Prevalansı”, “HBV Prevalence During Pregnancy”, “Gebelikte HCV Prevalansı”, “HCV Prevalence During Pregnancy”, “Gebelikte HSV Prevalansı”, “HSV Prevalence During Pregnancy”, “Gebelikte Viral Enfeksiyonlar”, “Doğum Öncesi Enfeksiyon Tarama Testleri”

## Appendix C

**Table A2:** Meta regression results and covariances of subgroups.

a. CMV							
Covariate	Coefficient	Standard Error	95% Lower	95% Upper	t-Value	2-Sided *p* Value	Set
Intercept	−475.6107	267.5159	−1029.0095	77.7881	−1.78	0.0886	F = 0.78 df = 6, 23 *p* = 0.5976
Publication quality score	3.2225	1.9247	−0.5498	6.9948	1.67	0.0941	
Sample size	−0.2362	0.4285	−1.1226	0.6502	−0.55	0.5868	
Region	0	0.0001	−0.0003	0.0002	−0.09	0.9258	
Method	−0.1324	0.1632	−0.4701	0.2053	−0.81	0.4256	
Year	0.0938	0.3334	−0.5959	0.7836	0.28	0.7809	
STATISTIC FOR THIS MODEL							
Test of this model: Simultaneous test that all coefficients (excluding intercept) are zero							
F = 0.78, df = 6, 23, *p* = 0.5976							
Goodness of fit: Test that unexplained variance is zero							
Tau² = 2.9003, Tau = 1.7030, I² = 99.40%, Q = 3818.31, df = 23, *p* = 0.0000							
COMPARISON OF THIS MODEL 1 WITH THE NULL MODEL							
Total between-study variance (intercept only)							
Tau² = 2.6512, Tau = 1.6283, I² = 99.71%, Q = 10143.37, df = 29, *p* = 0.0000							
Proportion of total between-study variance explained by this model							
R² analog = 0.00							
**b. HBV**							
**Covariate**	**Coefficient**	**Standard Error**	**95% Lower**	**95% Upper**	**t-value**	**2-sided *p* value**	**Set**
Intercept	109.9536	164.8442	−243.602	463.5093	0.67	0.5156	F = 0.82 df = 6, 14 *p* = 0.5702
Publication quality score	0.377	0.2329	−0.1226	0.8766	1.62	0.1279	
Sample size	0	0	0	0	−0.62	0.5422	
Region	−0.0286	0.0934	−0.2288	0.1716	−0.31	0.764	
Method	−0.0135	0.1854	−0.4112	0.3841	−0.07	0.9428	
Year	0.4829	0.7914	−1.2145	2.1804	0.61	0.5515	
STATISTIC FOR THIS MODEL							
Test of this model: Simultaneous test that all coefficients (excluding intercept) are zero							
F = 0.82, df = 6.14, *p* = 0.5702							
Goodness of fit: Test that unexplained variance is zero							
Tau² = 0.4703, Tau = 0.6858, I² = 97.33%, Q = 524.46, df = 14, *p* = 0.0000							
COMPARISON OF THIS MODEL 1 WITH THE NULL MODEL							
Total between-study variance (intercept only)							
Tau² = 0.4472, Tau = 0.6688, I² = 98.71%, Q = 1547.81, df = 20, *p* = 0.0000							
Proportion of total between-study variance explained by this model							
R² analog = 0.00							
**c. HCV**							
**Covariate**	**Coefficient**	**Standard Error**	**95% Lower**	**95% Upper**	**t-value**	**2-sided *p* value**	**Set**
Intercept	573.2131	526.6328	−672.0756	1818.5018	1.09	0.3124	F = 0.74 df = 6, 7 *p* = 0.6347
Publication quality score	−0.2235	0.3553	−1.0637	0.6167	−0.63	0.5493	
Sample size	0	0	0	0	−0.12	0.9055	
Region	0.2562	0.2125	−0.2464	0.7588	1.21	0.2672	
Method	−0.5524	0.7504	−2.3268	1.222	−0.74	0.4855	
Year	1.3451	1.716	−2.7127	5.4028	0.78	0.4588	
STATISTIC FOR THIS MODEL							
Test of this model: Simultaneous test that all coefficients (excluding intercept) are zero							
F = 0.74, df = 6, 7, *p* = 0.6347							
Goodness of fit: Test that unexplained variance is zero							
Tau² = 0.5784, Tau = 0.7605, I² = 79.98%, Q = 34.96, df = 7, *p* = 0.0000							
COMPARISON OF THIS MODEL 1 WITH THE NULL MODEL							
Total between-study variance (intercept only)							
Tau² = 0.5127, Tau = 0.7160, I² = 93.57%, Q = 202.29, df = 13, *p* = 0.0000							
Proportion of total between-study variance explained by this model							
R² analog = 0.00							
**d. Rubella**							
**Covariate**	**Coefficient**	**Standard Error**	**95% Lower**	**95% Upper**	**t-value**	**2-sided *p* value**	**Set**
Intercept	25.0864	136.7496	−256.5547	306.7275	0.18	0.8559	F = 1.21 df = 6, 25 *p* = 0.3319
Publication quality score	−0.24	0.242	−0.7385	0.2585	−0.99	0.331	
Sample size	−0.0002	0.0001	−0.0003	0	−2.44	0.0221 *	
Region	−0.0251	0.0893	−0.2091	0.1588	−0.28	0.7805	
Method	−0.174	0.1895	−0.5643	0.2162	−0.92	0.3671	
Year	0.3311	0.5971	−0.8986	1.5609	0.55	0.5841	
STATISTIC FOR THIS MODEL							
Test of this model: Simultaneous test that all coefficients (excluding intercept) are zero							
F = 1.21, df = 6, 25, *p* = 0.3319							
Goodness of fit: Test that unexplained variance is zero							
Tau² = 0.8308, Tau = 0.9115, I² = 99.58%, Q = 5967.23, df = 25, *p* = 0.0000							
COMPARISON OF THIS MODEL 1 WITH THE NULL MODEL							
Total between-study variance (intercept only)							
Tau² = 0.8597, Tau = 0.9272, I² = 99.80%, Q = 15335.60, df = 31, *p* = 0.0000							
Proportion of total between-study variance explained by this model							
R² analog = 0.03							

* *p* < 0.05.
